# Omission of adjuvant radiotherapy in low-risk elderly males with breast cancer

**DOI:** 10.1007/s12282-024-01560-y

**Published:** 2024-03-20

**Authors:** Kim Vo, Colton Ladbury, Stephanie Yoon, Jose Bazan, Scott Glaser, Arya Amini

**Affiliations:** 1 College of Osteopathic Medicine of the Pacific, Western University of Health Sciences, 309 E 2 ndSt, Pomona, CA 91766 USA; 2 Department of Radiation Oncology, City of Hope National Medical Center, 1500 East Duarte Road, Duarte, CA 91010 USA

**Keywords:** Male breast cancer, Radiation therapy, Breast neoplasm, Adjuvant therapy, Systemic therapy

## Abstract

**Purpose:**

Randomized clinical trials demonstrate that lumpectomy + hormone therapy (HT) without radiation therapy (RT) yields equivalent survival and acceptable local–regional outcomes in elderly women with early-stage, node-negative, hormone-receptor positive (HR +) breast cancer. Whether these data apply to men with the same inclusion criteria remains unknown.

**Methods:**

The National Cancer Database was queried for male patients ≥ 65 years with pathologic T1-2N0 (≤ 3 cm) HR + breast cancer treated with breast-conserving surgery with negative margins from 2004 to 2019. Adjuvant treatment was classified as HT alone, RT alone, or HT + RT. Male patients were matched with female patients for OS comparison. Survival analysis was performed using Cox regression and Kaplan − Meier method. Inverse probability of treatment weighting (IPTW) was applied to adjust for confounding.

**Results:**

A total of 523 patients met the inclusion criteria, with 24.4% receiving HT, 16.3% receiving RT, and 59.2% receiving HT + RT. The median follow-up was 6.9 years (IQR: 5.0–9.4 years). IPTW-adjusted 5-yr OS rates in the HT, RT, and HT + RT cohorts were 84.0% (95% CI 77.1–91.5%), 81.1% (95% CI 71.1–92.5%), and 93.0% (95% CI 90.0–96.2%), respectively. On IPTW-adjusted MVA, relative to HT, receipt of HT + RT was associated with improvements in OS (HR: 0.641; *p* = 0.042). RT alone was not associated with improved OS (HR: 1.264; *p* = 0.420).

**Conclusion:**

Among men ≥ 65 years old with T1-2N0 HR + breast cancer, RT alone did not confer an OS benefit over HT alone. Combination of RT + HT demonstrated significant improvements in OS. De-escalation of treatment through omission of either RT or HT at this point should be done with caution.

**Supplementary Information:**

The online version contains supplementary material available at 10.1007/s12282-024-01560-y.

## Introduction

Breast cancer in men is a rare age-related disease making up 1% of all breast cancers [1]. Although the lifetime risk of breast cancer is 1:1000 for a man compared to 1:8 for a woman, both sexes share overlapping risk factors leading to carcinogenesis [2, 3]. The risk of developing breast cancer increases with age, radiation exposure, conditions associated with high ratio of estrogen to androgen [4–9], family history of breast cancer [10], and established mutations associated with breast cancer. Owing to the rarity of the disease, there is low public awareness and an absence of screening programs leading to a later age of breast cancer diagnosis in men than women [3]. There is also a paucity of data to definitively guide how male breast cancer treatment should differ from treatment of female breast cancer, if at all.

Many clinical trials of breast cancer treatments have either excluded men or failed to enroll men; thus, treatment recommendations have been extrapolated from the results of female cohorts or data from cohorts of male patients treated at single institutions. The national treatment guidelines developed for women with early-stage breast cancer recommend mastectomy or breast-conserving therapy (lumpectomy + adjuvant radiotherapy). Despite breast-conserving surgery (BCS) plus radiotherapy having equivalent survival rates to mastectomy, due to anatomic considerations, most men (including those with early-stage disease) undergo mastectomy with either axillary lymph node dissection or sentinel-node biopsy without adjuvant radiation therapy (RT) [11, 12]. When they do undergo BCS, SEER data from 1988 to 2012 indicate that only 42% of men with early-stage I breast cancer received radiotherapy after breast-conserving surgery [12]. International data also show similar trends from 1990 to 2010 with almost 50% of men treated with (BCS) did not receive RT [13].

Efforts of de-intensifying breast cancer treatment by omitting RT in elderly patients undergoing BCS have increased over the last decade due to the improvements in breast imaging, surgical techniques, and integration of hormone therapy (HT). Because a greater proportion of men relative to women with breast cancer are hormone receptor-positive, standard adjuvant tamoxifen is recommended postoperatively and has shown benefits in patients with hormone-positive tumors after lumpectomy with negative margins [14–16]. The recent randomized clinical trials demonstrated BCS + hormone therapy without RT yields equivalent survival and acceptable local–regional outcomes in elderly women with early-stage, node-negative (T1-2N0) hormone-receptor positive (HR +) breast cancer [17, 18]. Whether these data apply to men with the same inclusion criteria remains unknown. However, it is certainly an attractive option due to the hormone positivity that is common in this patient population.

Although breast cancer in men is rare, its incidence has increased globally over the last few decades [19]. There has been significant progress in the understanding of the molecular and pathology of the disease; however, optimal management of male breast cancer remains understudied. Due to the lack of research and clinical trials in treatment regimen outcomes using male cohorts, many gaps remain in our knowledge, and it is unknown if treatment deintensification efforts such as RT omission is a preferred option for elderly male patients with early-stage breast cancer. Extrapolating the results from recent randomized clinical trials studying treatment outcomes of low-grade breast cancer in elderly females with lumpectomy + HT and without RT, we hypothesized that outcomes in males would be comparable to those seen in females, with RT not conferring an overall survival (OS) benefit over HT alone. Herein, we performed a retrospective analysis of the impact of adjuvant treatment options on survival outcomes in men, who would meet the criteria for RT omission based on the existing randomized trial inclusion criteria.

## Materials and methods

### Data source

The National Cancer Database (NCDB) is a joint project of the American College of Surgeons and the American Cancer Society that compiled hospital cancer registry data from over 1500 commission-accredited facilities contributing approximately 75% of cancer programs in the United States [20]. The NCDB contains detailed information on patients initially diagnosed with cancer, disease stage, risk factors, and receipt of treatment at a Commission on Cancer accredited facilities detailing surgery, radiation, and chemotherapy delivered during the first course of treatment. The NCDB has established criteria to ensure the data submitted is identified and meets specific quality benchmarks. The American College of Surgeons and the Commission on Cancer have not verified and are not responsible for the analytic or statistical methodology used or for the conclusions drawn from these data by the investigators. Our study was considered exempt from institutional review board review.

### Patient selection

**Fig. 1:**
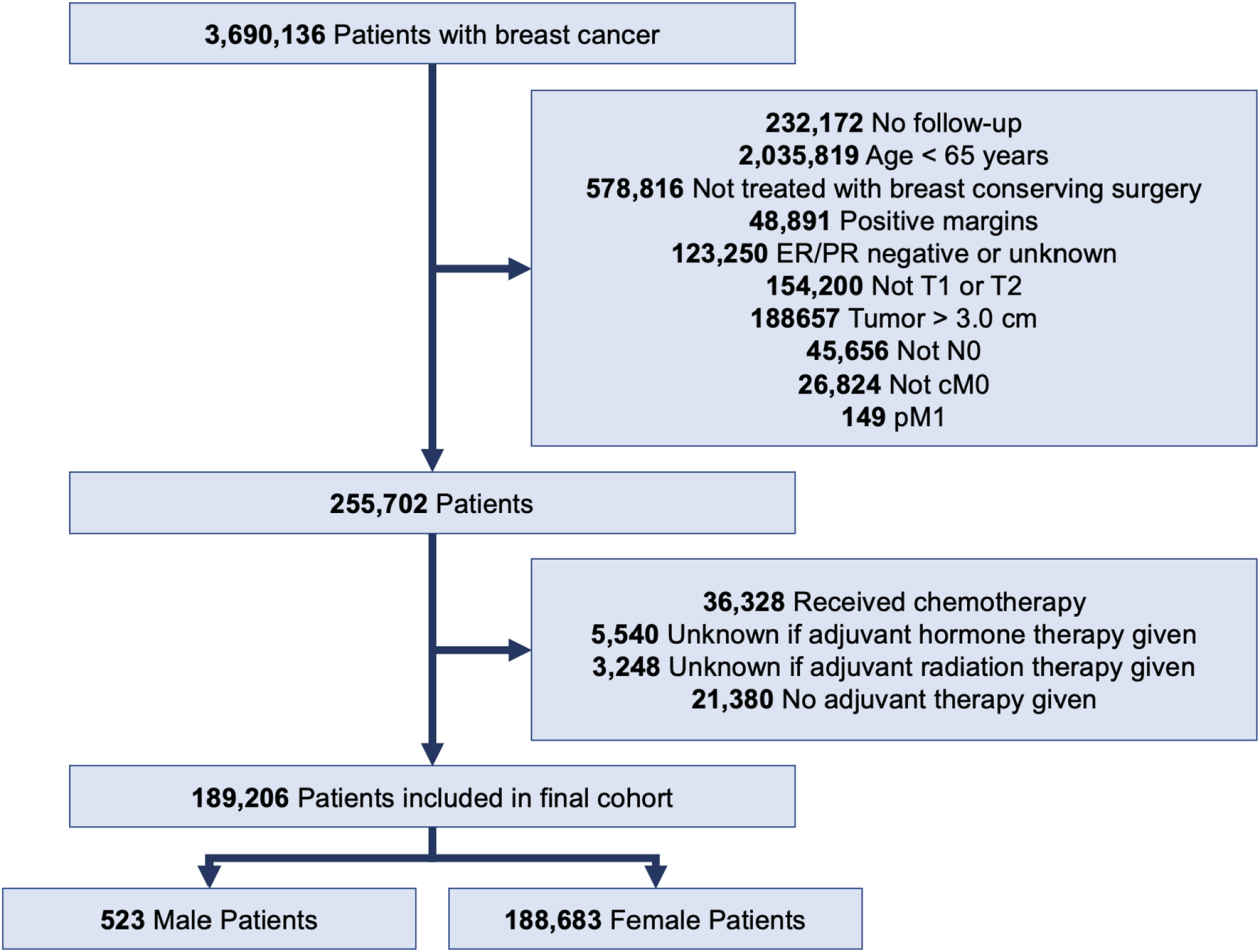
Patient selection schema for male and female cohorts

The NCDB was queried for patients ≥ 65 years with pathologic T1-2N0 (≤ 3 cm) HR + breast cancer treated with breast-conserving surgery with negative margins from 2004 to 2019. Patients who received chemotherapy, had nodal or distant metastases, or had unknown follow-ups were excluded. The full patient selection is shown in Fig. 1. In these patients, adjuvant treatment was classified as HT alone, RT alone, or HT + RT.

### Statistical analyses

Descriptive statistics of the overall cohort were generated. Patients receiving a specific type of adjuvant treatment were compared using the *χ*^2^ for categorical data and the Wilcoxon Rank Sum test for continuous variables. Predictors of receiving a given adjuvant therapy were characterized using multivariable logistic regression with backward selection. Survival analysis was performed using Cox regression and Kaplan − Meier analysis. To adjust for confounding, inverse probability of treatment weighting (IPTW) was used, employing significant features identified in the logistic regression. Owing to limitations of survival analysis on retrospective data, male patients were also matched with female patients to determine comparable outcomes based on year of diagnosis (± 2 years), age (± 4 years), Charlson − Deyo comorbidity score, T-stage, grade, and adjuvant treatment. For all analyses, statistical significance was defined as p < 0.05. All statistical analyses were performed using open-source libraries in Python 3.10 (Python Software Foundation, Wilmington, DE, USA) and R 4.2.2 (The R Foundation, Indianapolis, IN, USA).

## Results

A total of 523 male patients met the inclusion criteria, with 24.4%, 16.3%, and 59.2% receiving adjuvant HT, RT, and HT + RT, respectively. This is in comparison to the female cohort, where 18.6%, 13.4%, and 67.9% received HT, RT, and HT + RT, respectively. Males who received HT + RT had a median age of 71 years compared to 76.5 for HT (*p* = 0.008) and 75 for RT (*p* < 0.001). One of ten (10%) HER2 + males and 303/3563 (8.5%) of HER2 + females received some form of immune targeted therapy including HER2-directed therapies. Descriptive statistics for the male cohort are shown in Table 1. Descriptive statistics for the female cohort are shown in Supplemental Table 1.

**Table 1:** Descriptive statistics of male cohort

Characteristic	All patients (*N* = 523)	Adjuvant therapy			*p*		
		HT + RT (*N* = 310)	HT (*N* = 128)	RT (*N* = 85)	HT + RT vs HT	HT + RT vs RT	HT vs RT
Year of diagnosis [median (range)]	2011 (2004–2019)	2011 (2004–2018)	2013 (2004–2019)	2009 (2004–2018)	0.008	< 0.001	< 0.001
Age [median (range)]	73.0 (65–90)	71.0 (65–88)	76.5 (65–90)	75.0 (65–90)	< 0.001	0.003	0.107
Race and ethnicity					0.943	0.661	0.631
Asian/Pacific islander	8 (1.5%)	5 (1.6%)	1 (0.8%)	2 (2.4%)			
Black	39 (7.5%)	20 (6.5%)	10 (7.8%)	9 (10.6%)			
Hispanic	6 (1.1%)	4 (1.3%)	2 (1.6%)	0 (0.0%)			
Other	2 (0.4%)	1 (0.3%)	1 (0.8%)	0 (0.0%)			
Unknown	5 (1.0%)	3 (1.0%)	1 (0.8%)	1 (1.2%)			
White	463 (88.5%)	277 (89.4%)	113 (88.3%)	73 (85.9%)			
Charlson − Deyo score					0.031	0.338	0.093
0	406 (77.6%)	247 (79.7%)	89 (69.5%)	70 (82.4%)			
1	87 (16.6%)	43 (13.9%)	31 (24.2%)	13 (15.3%)			
2 +	30 (5.7%)	20 (6.5%)	8 (6.2%)	2 (2.4%)			
Tumor laterality					0.281	0.964	0.37
Left	246 (47.0%)	150 (48.4%)	54 (42.2%)	42 (49.4%)			
Right	277 (53.0%)	160 (51.6%)	74 (57.8%)	43 (50.6%)			
pT					0.226	0.14	0.027
T1	458 (87.6%)	272 (87.7%)	106 (82.8%)	80 (94.1%)			
T2	65 (12.4%)	38 (12.3%)	22 (17.2%)	5 (5.9%)			
Tumor size [median (range)]	11.0 (2–30)	11.0 (2–30)	12.0 (2–30)	10.0 (2–28)	0.328	0.213	0.076
Histology					0.714	0.909	0.587
IDC	404 (77.2%)	239 (77.1%)	100 (78.1%)	65 (76.5%)			
ILC	29 (5.5%)	18 (5.8%)	5 (3.9%)	6 (7.1%)			
Other	90 (17.2%)	53 (17.1%)	23 (18.0%)	14 (16.5%)			
ER					1	0.904	0.836
Negative	2 (0.4%)	1 (0.3%)	0 (0.0%)	1 (1.2%)			
Positive	521 (99.6%)	309 (99.7%)	128 (100.0%)	84 (98.8%)			
PR					0.681	< 0.001	0.004
Negative	37 (7.1%)	13 (4.2%)	7 (5.5%)	17 (20.0%)			
Positive	483 (92.4%)	296 (95.5%)	120 (93.8%)	67 (78.8%)			
Unknown	3 (0.6%)	1 (0.3%)	1 (0.8%)	1 (1.2%)			
HER2					0.034	< 0.001	< 0.001
Negative	336 (64.2%)	213 (68.7%)	89 (69.5%)	34 (40.0%)			
Positive	10 (1.9%)	3 (1.0%)	6 (4.7%)	1 (1.2%)			
Unknown	177 (33.8%)	94 (30.3%)	33 (25.8%)	50 (58.8%)			
Grade					0.991	0.835	0.808
Grade 1	197 (38.9%)	116 (37.4%)	48 (37.5%)	33 (38.8%)			
Grade 2	238 (47.0%)	142 (45.8%)	55 (43.0%)	41 (48.2%)			
Grade 3	49 (9.7%)	29 (9.4%)	12 (9.4%)	8 (9.4%)			
Unknown	22 (4.3%)	14 (4.5%)	6 (4.7%)	2 (2.4%)			
LVSI					0.61	< 0.001	< 0.001
LVSI +	26 (5.0%)	18 (5.8%)	7 (5.5%)	1 (1.2%)			
LVSI-	295 (56.4%)	183 (59.0%)	82 (64.1%)	30 (35.3%)			
Unknown	202 (38.6%)	109 (35.2%)	39 (30.5%)	54 (63.5%)			

*HT* hormone therapy, *RT* radiation therapy, *IDC* intraductal carcinoma, *ILC* intralobular carcinoma, *ER* estrogen receptor, *PR* progesterone receptor, *HER2* human epidermal growth receptor 2, *LVSI* lymphovascular space invasion

On multivariable logistic regression, later years of diagnosis were associated with decreased odds of receiving RT alone (OR: 0.816; p < 0.001) and increased odds of receiving HT alone (OR: 1.182; *p* < 0.001). Later year of diagnosis was not a significant factor in receiving HT + RT. Charlson − Deyo score was only a significant factor in receiving HT alone (1 vs 0; OR: 1.998; *p* = 0.01). Patients with negative progesterone receptor (PR) status were more likely to receive RT alone (OR: 6.051; *p* < 0.001) and less likely to receive HT + RT (OR: 0.373; *p* = 0.009). Patients tested positive for human epidermal growth receptor 2 status (HER2) were more likely to receive HT alone (OR: 4.306; *p* = 0.036) and less likely to receive HT + RT (OR: 0.249; *p* = 0.050). Full logistic regressions are shown in Table 2.

**Table 2:** Multivariable logistic regression of features associated with adjuvant treatment decision

	RT Alone		HT		HT + RT	
Category	OR	p	OR	p	OR	p
Year of Diagnosis	0.816 (0.759–0.878)	< 0.001	1.182 (1.077–1.298)	< 0.001	–	–
Age	–	–	1.099 (1.063–1.137)	< 0.001	0.916 (0.89–0.944)	< 0.001
Charlson − Deyo score	–	–	–	–	–	–
0	–	–	Ref		–	–
1	–	–	1.998 (1.178–3.389)	0.01	–	–
2 +	–	–	0.933 (0.384–2.265)	0.879	–	–
PR	–	–	–	–	–	–
Positive	Ref		–	–	Ref	
Negative	6.051 (2.859–12.806)	< 0.001	–	–	0.373 (0.179–0.779)	0.009
Unknown	1.669 (0.146–19.092)	0.681	–	–	0.451 (0.039–5.227)	0.524
HER2	–	–	–	–	–	–
Negative	–	–	Ref		Ref	
Positive	–	–	4.306 (1.102–16.818)	0.036	0.249 (0.06–1.000)	0.05
Unknown	–	–	1.366 (0.683–2.73)	0.378	0.728 (0.493–1.075)	0.11

**Fig. 2:**
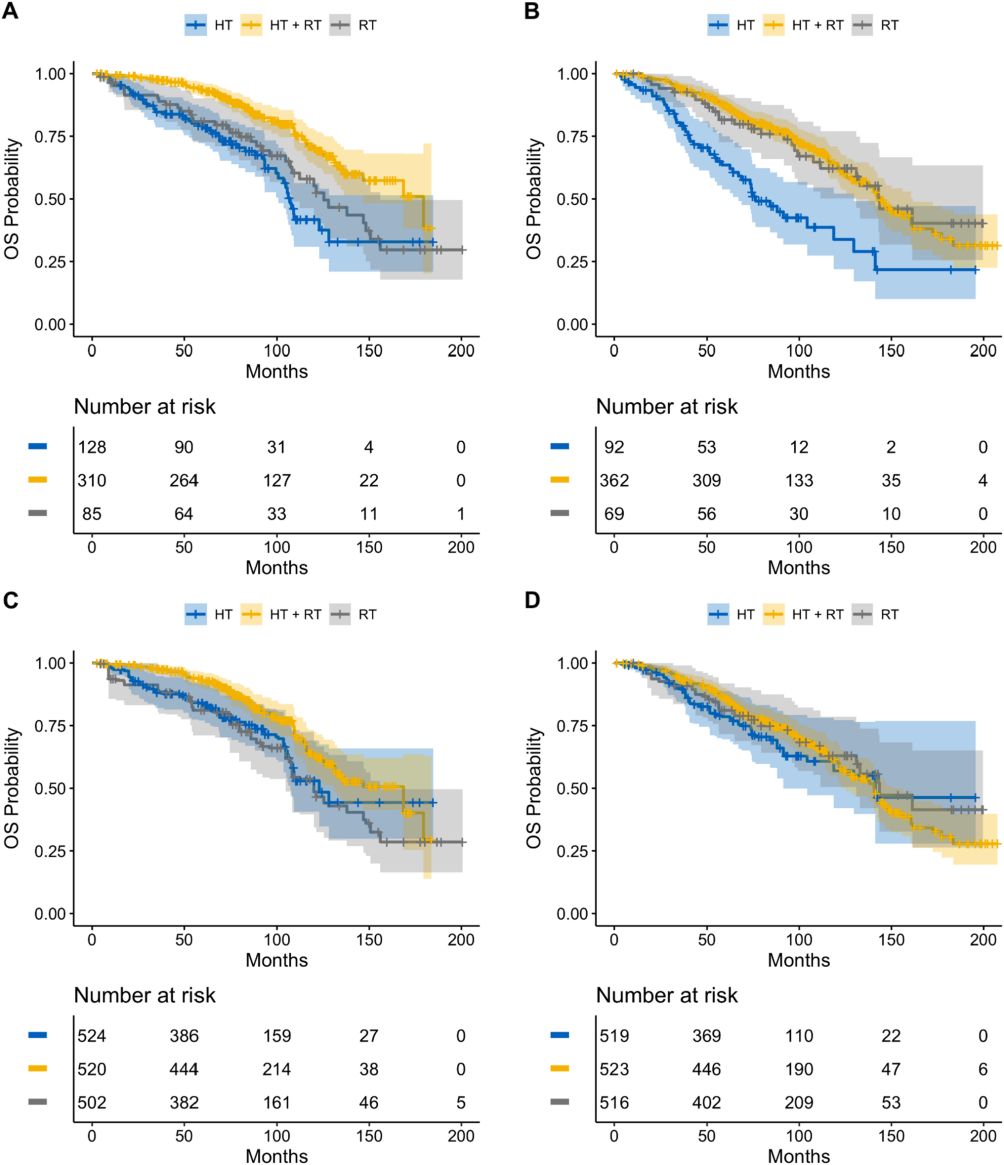
Unadjusted Kaplan Meier estimates of overall survival in male cohort (A) and female cohort (B); IPTW Kaplan Meier estimates of overall survival in male cohort (C) and female cohort (D)

Median follow-up in the male cohort was 6.9 years (IQR: 5.0–9.4 years). Unadjusted 5-yr OS rates in the HT, RT, and HT + RT cohorts were 79.2% (95% CI 70.7–85.5%), 80.9% (95% CI 70.3–88.0%), and 93.3% (95% CI 89.7–95.7%), respectively. IPTW-adjusted 5-yr OS rates in the HT, RT, and HT + RT cohorts were 84.0% (95% CI 77.1–91.5%), 81.1% (95% CI 71.1–92.5%), and 93.0% (95% CI 90.0–96.2%), respectively. For comparison, in the female cohort, adjusted 5-yr OS rates in the HT, RT, and HT + RT cohorts were 78.6% (95% CI 69.5–88.8%), 81.1% (95% CI 71.6–91.8%), and 85.9% (95% CI 82.0–89.9%), respectively. Kaplan − Meier estimates are shown in Fig. 2.

On both unadjusted (HR: 1.116; *p* = 0.633) and IPTW-adjusted Cox regression (HR: 1.264; *p* = 0.420), there was no significant difference in OS when comparing RT alone to HT alone. HT + RT was associated with improved OS on both unadjusted (HR: 0.603; *p* = 0.01) and IPTW-adjusted (HR: 0.641; *p* = 0.042) Cox regressions. In addition to the type of adjuvant therapy, age, Charlson − Deyo score, pT stage, and grade were all significant predictors of OS. On IPTW-adjusted Cox regression, tumor grade was no longer a significant predictor. Full Cox regressions in the male cohort are shown in Table 3. In the IPTW-adjusted Cox regression in the female cohort, adjuvant therapy, pT stage, and grade were not significant predictors of OS. The Cox regression in the female cohort is shown in Supplemental Table 2.

**Table 3:** Multivariable Cox proportional-hazards regression of OS in male cohort

	Unadjusted		IPTW	
Category	HR	*p*	HR	*p*
Age	1.112 (1.083–1.141)	< 0.001	1.09 (1.053–1.128)	< 0.001
Charlson − Deyo Score				
0	Ref		Ref	
1	1.718 (1.175–2.511)	0.005	1.655 (1.053–2.6)	0.029
2 +	1.644 (0.821–3.293)	0.161	3.289 (1.166–9.28)	0.024
pT				
T1	Ref		Ref	
T2	1.894 (1.231–2.913)	0.004	1.843 (1.008–3.367)	0.047
Grade				
Grade 1	Ref		Ref	
Grade 2	0.712 (0.497–1.019)	0.064	0.733 (0.462–1.163)	0.187
Grade 3	2.076 (1.275–3.38)	0.003	1.757 (0.958–3.224)	0.068
Unknown	0.797 (0.374–1.697)	0.556	0.886 (0.414–1.897)	0.756
Adjuvant therapy				
HT	Ref		Ref	
HT + RT	0.603 (0.41–0.888)	0.01	0.641 (0.418–0.983)	0.042
RT	1.116 (0.71–1.755)	0.633	1.264 (0.716–2.231)	0.420

*HT* hormone therapy, *RT* radiation therapy

## Discussion

In this study, men > 65 yo with early-stage T1-T2N0M0 breast cancer treated with adjuvant therapies, the IPTW adjusted 5-year OS after BCS was significantly higher among male patients who received both HT + RT than HT or RT alone. Notably, this finding was not observed in the female cohort. Our data suggests there may be a meaningful additive benefit of HT + RT, and therefore RT omission in this group of patients should be done with caution and shared decision making absent higher quality data. The observed difference in male breast cancer (MBC) may be due to the differences in breast cancer treatment guidelines and adherence, aggressive behavior of the primary tumor, differences in anatomy impact outcomes, adjuvant hormone therapy adherence, and possible uncontrolled confounders.

The primary tumor in men has more aggressive features. A large NCDB study evaluating gender differences in breast cancer found breast cancers in men were one-third larger than women (20.0 vs. 15.0 mm mean size, *p* < 0.00001), less likely to be grade 1 (16.0% vs. 20.7%, *p* < 0.0001), and more likely to have nodal metastasis (41.9% vs. 33.2%, *p* < 0.001). The investigators argued that the differences in patient age, tumor size, and stage at presentation could represent a lead time bias when diagnosing breast cancer since female breast cancer (FBC) was discovered through routine mammographic screening. Nevertheless, compared to women, men with estrogen receptor positive (ER +) breast cancer particularly have worse OS after adjusting for age, race, clinical and treatment characteristics, and access to care [21, 22]. Another NCDB cohort study (2004–2014) led by Wang et al. also found that men had worse survival than women across all cancer stages [22]. This finding is further supported by a SEER-based study that showed a lower 5-year stage-by-stage OS for male and female cases [23]. Some studies suggest sex disparity in OS was only evident in early-stage disease as observed in our study, but not for advanced-stage disease [21, 24, 25], while other studies have found no significant differences [26, 27]. Despite the inconsistent results on stage-specific survival, it is important to note that diagnostic approaches and treatment strategies have improved based on the results from female data, which could have led to some result variability observed in male patients. Regardless, the existing data support MBC being comparatively more aggressive than female breast cancer, meaning extrapolation of certain treatment options, particularly de-escalation options, should be done with caution.

Although treatment strategies for MBC have followed the guidelines developed from female breast cancer trials, treatment guidelines in men undergoing BCS lagged behind the current literature. Previously, in women with early-stage breast cancer, the mainstay treatment consisted of mastectomy or breast-conserving therapy adjuvant HT [28]. Recent clinical trials, Cancer and Leukemia Group B9343 cooperative group (CALGB 9343) and Postoperative Radiotherapy in Minimum-risk Elderly (PRIME II), have shown that older females with low grade, early-stage breast cancer who received both HT + RT shown significant improvement in local control and subsequent risk of future relapse [18, 29]. Despite the improvement in local control with RT, there was no significant difference in distant metastasis or OS. These findings substantiated a modification in the current clinical practice guideline, which allows omission of RT after BCS in women with T1N0, HR + early breast cancer who plan to complete a 5-year course of HT [30]. However, for most men with early-stage breast cancer, breast conservation is not common as men have small amounts of breast tissue. As such, only 18% of men with T1N0 tumors underwent BCS [31]. These men are also less likely to receive lymph node staging after accounting for differences in age, race, tumor stage, grade, and year of diagnosis [32]. Perhaps, the lack of published treatment guidelines specific to MBC has led to noncompliance with guideline norms, leading to less nodal exploration and insufficient radiation [33]. Bakalov et al. found that RT was associated with a mortality reduction of 70% in the propensity-matched model; however, it was omitted in a third of BCS cases in men. This could be attributed to the under-implementation of guidelines despite RT being a standard of care [34]. It also adds to the body of evidence that supports the use of RT after BCS as this was associated with a mortality reduction of 70%. Besides the omission of RT, HT is also under-utilized despite its effectiveness in MBC. Venigalla et al. found that over 33% of male patients with HR-positive disease did not receive HT and that men received HT less frequently than women did, indicating possible sex disparity in care [35]. Their data suggested that the underutilization may be explained by the lack of evidence-based guidelines for MBC management.

Another factor that could possibly improve OS was the prognostic significance of ER status in regression analyses. Men with ER + positive breast cancer had a 30% reduction in the risk of death as these patients are more responsive to HT than those with ER- breast cancer [36]. However, in both sexes, the difference between ER + and ER- appears to fade with follow-ups of more than 7.5 years. This difference is more exaggerated in MBC suggesting possible inadequacy of HT compliance in men and potential biological differences between men and women. Many studies have reported suboptimal adherence to tamoxifen in men compared with women [37–39]. A meta-regression analysis estimated adherence in men to vary from 64.6% (95% CI 47.8–77.2) to 79.2% (95% CI 67.5–87.0) for tamoxifen treatment [38]. In another study using the SEER-Medicare database from 2007 to 2013, Oke et al. [39] found that among elderly men, 65 years and older, 48.3% had discontinued tamoxifen within 5 years. They also found that those having higher comorbidities and a higher age of diagnosis (> 80 years) are at greater risk of discontinuing hormone treatment early. This could be due to lower tolerability of side effects of tamoxifen [39, 40], which might also contribute to why the HT arm was worse than HT + RT in our study. In a retrospective analysis of tamoxifen-related side effects in MBC patients who were on tamoxifen, Pemmaruju et al. found that over 20% of patients discontinued tamoxifen due to side effects (e.g. weight gain and sexual dysfunction) with a median time of discontinuation of 49 months [41]. Out of those patients, 31% were physician-directed due to an increase in thromboembolic events (VTEs), while 69% were patient-directed based on the intolerable side effects. Given survival benefit from hormone therapy is dependent on regular usage and treatment duration, repeated deviations from the prescribed course may reduce efficacy and result in poorer outcomes [37].

It is important to note that even in the absence of an overall survival benefit, the role of RT should not be trivialized, as local recurrences can certainly be impactful if they ultimately require additional treatment including surgery, further endocrine therapy, or chemotherapy, which can all contribute to clinically meaningful treatment morbidity [42, 43]. Importantly, due to the limitations in the NCDB, oncologic outcomes beyond OS, including local recurrences, are unavailable, so our data is unable to characterize how different adjuvant treatment regimens impact disease control specifically. Further, the morbidity associated with endocrine therapy has significant implications for quality of life and also can increase the risk of nononcologic conditions such as heart disease [44]. Therefore, in MBC, one could argue it might be reasonable to instead consider the omission of endocrine treatment, particularly in the era of hypofractionated and ultra-hypofractionated RT, and the greater benefit conferred by RT compared to HT in women based on randomized data [43, 45]. Such a concept is being investigated in the ongoing EUROPA trial, though this will not provide clarity for MBC given that males are excluded from enrollment [46]. Despite the lack of male enrollment in breast cancer clinical trials, recent studies have included men. NRG-BR007, a phase III randomized clinical trial is looking to evaluate whether de-escalation of breast radiation for stage I, hormone positive, HER-, underwent lumpectomy with Oncotype DX RS ≤ 18 is appropriate [47]. The study is still undergoing patient accrual.

In FBC, Oncotype DX recurrence scoring (RS) system has been used widely in early-stage, ER + , node-negative breast tumors to risk stratify tumor recurrence for chemotherapy. The prognostic value of Oncotype RS in men remains understudied. A couple of retrospective database studies have shown Oncotype RS is prognostic for breast cancer specific-survival and OS among male patients with N0-1 disease. These studies have also found comparable RS in both sexes; however, the frequency of low (RS < 10) and high RS (> 31) are higher in men compared to women respectively [22, 48]. It was noted that very low RS subtype was found in older men and high RS subtypes in younger men (< 40 years), which further suggests that there might be biologically distinct ER + disease subtypes defined by RS results [48]. An NCDB analysis with 848 men with stage I − II and N0 − N1 breast cancer diagnosed from 2010 to 2014 showed an association of RS with OS, with 5-year OS of 97%, 91%, and 83% for RS < 10, 11–25, and ≥ 26, respectively (*p* = 0.003) [22]. After adjusting for demographic and clinical characteristics except chemotherapy, male patients with RS ≥ 11 had higher mortality risk compared to those with RS < 10.

Another analysis of 322 male patients with breast cancer from SEER database diagnosed between 2004 and 2012 also reported a trend in increasing RS risk (< 18, 18–30, and ≥ 31) is associated with lower 5-yr breast cancer specific survival (99%, 96%, and 81%, respectively) and 5-yr OS (93%, 86%, and 70%, respectively) [48]. In the absence of validation studies, the comparisons drawn from the retrospective studies have shown that RS was positively associated with mortality in male patients compared to female counterparts. It is not unreasonable to utilize the combination of adjuvant chemotherapy and hormone therapy in male patients with Oncotype scores > 26 regardless of node status, which is consistent with current recommendations for females with RS > 26 [49].

Our study has additional limitations typical of retrospective data analysis using a large national database. The NCDB does not provide detailed data regarding the duration of HT treatment, which is an important metric in clinical trials. Additionally, our cohort includes patients diagnosed between 2004 and 2019; however, the NCDB did not reliably account for HER2 status until 2010. Although we excluded patients who received chemotherapy, we did not exclude those who received immunotherapy or had HER2 status. The possible influence of unmeasured confounders related to selection bias, which we tried to mitigate by including all potential confounders in the multivariate model, allowing truly independent variables to appear statistically significant. Furthermore, due to limitations of survival analysis on retrospective data, male patients were also matched with female patients to determine comparable outcomes based on year of diagnosis (± 2 years), age (± 4 years), Charlson − Deyo comorbidity score, T-stage, grade, and adjuvant treatment. However, even after utilizing statistical methods to reduce confounding, it is impossible to account for all potential confounders that might affect this study.

The precise role of RT in treating breast cancer in men, who undergo BCS, is uncertain as other studies have failed to show a survival advantage with this modality. There is a strong need for trials that focus on MBC to capture the differences and effective impact of interventions on health-related quality of life and outcomes. It is possible that MBC should be considered a unique disease, rather than being considered analogous to FBC. The development of treatment guidelines for MBC driven by data collected from studies that include male participants would be beneficial for this population cohort.

## Conclusions

Among men ≥ 65 years old with T1-2N0 HR + breast cancer, RT alone did not confer an OS benefit over HT alone. Combined RT + HT did yield improvements in OS, though there are likely significant unmeasured confounders contributing to these outcomes in patients treated with the most aggressive approach. Though not unreasonable, de-escalation of treatment through omission of either RT or HT at this point should be done with caution and with shared decision-making with patients. Owing to its rarity, a randomized trial in male breast cancer is unlikely, but optimally future multi-institutional cohort or prospective studies might further elucidate the optimal adjuvant treatment of MBC treated with BCS.

## Conflict of interest

The authors have no relevant conflicts of interest to report.

### Supplementary Information

Below is the link to the electronic supplementary material.

Supplementary file1 (DOCX 20 KB)

## Data Availability

The data that support the findings of this study are available from the National Cancer Database (NCDB) supported by the American College of Surgeons. Data may be requested through the NCDB request portal (https://ncdbapp.facs.org/puf/).

## References

[1] Siegel RL, Miller KD, Jemal A (2018). Cancer statistics, 2018. CA Cancer J Clin.

[2] Wan A, Zhang G, Ma D, Zhang Y, Qi X (2023). An overview of the research progress of BRCA gene mutations in breast cancer. Biochim Biophys Acta BBA Rev Cancer.

[3] Constantinou N, Marshall C, Marshall H (2023). Discussion and optimization of the male breast cancer patient experience. J Breast Imaging.

[4] Tahrir Y, Bertal A, Mawhoub S, Makhchoune M, Ibahiouin K, Lakhdar A (2022). A cerebellopontine angle metastatis of a male breast cancer: case report. Ann Med Surg..

[5] Pant VP, Dallakoti N, Pokhrel N, Chaudhary S, Dulal S, Paudyal P (2022). Metastases after mastectomy for ductal carcinoma in situ of the male breast; a case report. Ann Med Surg..

[6] Fang W, Huang Y, Han X, Peng J, Zheng M (2022). Characteristics of metastasis and survival between male and female breast cancer with different molecular subtypes: a population-based observational study. Cancer Med.

[7] de Carvalho TP, de Oliveira AR, dos Santos DO (2022). Histopathologic and immunophenotypic profile of a spontaneous mammary carcinoma in a male Humboldt’s white-fronted capuchin (Cebus albifrons). J Med Primatol.

[8] Oke O, Niu J, Chavez-MacGregor M, Zhao H, Giordano SH (2022). Adjuvant tamoxifen adherence in men with early-stage breast cancer. Cancer.

[9] Almansour NM (2022). Triple-negative breast cancer: a brief review about epidemiology, risk factors, signaling pathways, treatment and role of artificial intelligence. Front Mol Biosci..

[10] Appiah D, Mai M, Parmar K (2022). A prospective population-based study of cardiovascular disease mortality following treatment for breast cancer among men in the United States, 2000–2019. Curr Oncol.

[11] Masuda N, Kosaka N, Iwata H, Toi M (2021). Palbociclib as an early-line treatment for Japanese patients with hormone receptor–positive/human epidermal growth factor receptor 2–negative advanced breast cancer: a review of clinical trial and real-world data. Int J Clin Oncol.

[12] Zelli V, Silvestri V, Valentini V (2021). Transcriptome of male breast cancer matched with germline profiling reveals novel molecular subtypes with possible clinical relevance. Cancers.

[13] Liu FC, Veierød MB, Kjærheim K (2021). Excess risk of male breast cancer in the Norwegian offshore petroleum workers (NOPW) cohort: a possible link to extreme night shift work?. Breast Cancer Res.

[14] Amer M, Vaccalluzzo L, Vena W, Mazziotti G, Morenghi E, Pizzocaro A (2023). Oncological diseases in Klinefelter syndrome: an overview. Minerva Endocrinol.

[15] Noe JF, Bush MA (2021). Endocrine therapy for breast cancer. J Nurse Pract.

[16] Kraus AL, Yu-Kite M, Mardekian J (2022). Real-world data of palbociclib in combination with endocrine therapy for the treatment of metastatic breast cancer in men. Clin Pharmacol Ther.

[17] Buszek SM, Lin HY, Bedrosian I (2019). Lumpectomy plus hormone or radiation therapy alone for women aged 70 years or older with hormone receptor-positive early stage breast cancer in the modern era: an analysis of the national cancer database. Int J Radiat Oncol Biol Phys.

[18] Kunkler IH, Williams LJ, Jack WJL, Cameron DA, Dixon JM (2023). Breast-conserving surgery with or without irradiation in early breast cancer. N Engl J Med.

[19] Chen Z, Xu L, Shi W (2020). Trends of female and male breast cancer incidence at the global, regional, and national levels, 1990–2017. Breast Cancer Res Treat.

[20] Bilimoria KY, Stewart AK, Winchester DP, Ko CY (2008). The national cancer data base: a powerful initiative to improve cancer care in the United States. Ann Surg Oncol.

[21] Greif JM, Pezzi CM, Klimberg VS, Bailey L, Zuraek M (2012). Gender differences in breast cancer: analysis of 13,000 breast cancers in men from the national cancer data base. Ann Surg Oncol.

[22] Wang F, Shu X, Meszoely I (2019). Overall mortality after diagnosis of breast cancer in men vs women. JAMA Oncol.

[23] Giordano SH, Cohen DS, Buzdar AU, Perkins G, Hortobagyi GN (2004). Breast carcinoma in men. Cancer.

[24] Leone J, Zwenger AO, Leone BA, Vallejo CT, Leone JP (2019). Overall survival of men and women with breast cancer according to tumor subtype. Am J Clin Oncol.

[25] Wu SG, Zhang WW, Liao XL (2017). Men and women show similar survival outcome in stage IV breast cancer. The Breast.

[26] El-Tamer MB, Komenaka IK, Troxel A (2004). Men with breast cancer have better disease-specific survival than women. Arch Surg.

[27] Miao H, Verkooijen HM, Chia KS (2011). Incidence and outcome of male breast cancer: an international population-based study. J Clin Oncol Off J Am Soc Clin Oncol.

[28] Giordano SH (2018). Breast Cancer in Men. N Engl J Med.

[29] Hughes KS, Schnaper L, Berry D (2004). Lumpectomy plus tamoxifen with or without irradiation in women 70 years of age or older with early breast cancer. N Engl J Med.

[30] Gradishar WJ, Moran MS, Abraham J (2022). Breast cancer, version 3.2022, NCCN clinical practice guidelines in oncology. J Natl Compr Cancer Netw JNCCN..

[31] Leone JP, Leone J, Zwenger AO, Iturbe J, Leone BA, Vallejo CT (2017). Locoregional treatment and overall survival of men with T1a, b, cN0M0 breast cancer: a population-based study. Eur J Cancer.

[32] Cloyd JM, Hernandez-Boussard T, Wapnir IL (2013). Poor compliance with breast cancer treatment guidelines in men undergoing breast-conserving surgery. Breast Cancer Res Treat.

[33] Eisinger F, Ronda I, Puig B, Camerlo J, Giovannini MH, Bardou VJ (2007). Breast cancer guidelines—physicians’ intentions and behaviors. Int J Cancer.

[34] Bakalov V, Jayakrishnan TT, Abel S, Hilton C, Rusia B, Wegner RE (2021). The use of adjuvant radiation therapy in male breast cancer and its impact on outcomes. Cancer Treat Res Commun.

[35] Korde LA, Zujewski JA, Kamin L (2010). Multidisciplinary meeting on male breast cancer: summary and research recommendations. J Clin Oncol.

[36] Leone JP, Zwenger AO, Iturbe J (2016). Prognostic factors in male breast cancer: a population-based study. Breast Cancer Res Treat.

[37] Demissie S, Silliman RA, Lash TL (2016). Adjuvant Tamoxifen: Predictors of Use, Side Effects, and Discontinuation in Older Women. J Clin Oncol.

[38] Huiart L, Ferdynus C, Giorgi R (2013). A meta-regression analysis of the available data on adherence to adjuvant hormonal therapy in breast cancer: summarizing the data for clinicians. Breast Cancer Res Treat.

[39] Venigalla S, Carmona R, Guttmann DM (2018). Use and effectiveness of adjuvant endocrine therapy for hormone receptor-positive breast cancer in men. JAMA Oncol.

[40] Moredo Anelli TF, Anelli A, Tran KN, Lebwohl DE, Borgen PI (1994). Tamoxifen adminstration is associated with a high rate of treatment-limiting symptoms in male breast cancer patients. Cancer.

[41] Pemmaraju N, Munsell MF, Hortobagyi GN, Giordano SH (2012). Retrospective review of male breast cancer patients: analysis of tamoxifen-related side-effects. Ann Oncol.

[42] Blamey RW, Bates T, Chetty U (2013). Radiotherapy or tamoxifen after conserving surgery for breast cancers of excellent prognosis: British association of surgical oncology (BASO) II trial. Eur J Cancer.

[43] Fisher B, Bryant J, Dignam JJ (2002). Tamoxifen, radiation therapy, or both for prevention of ipsilateral breast tumor recurrence after lumpectomy in women with invasive breast cancers of one centimeter or less. J Clin Oncol Off J Am Soc Clin Oncol.

[44] Wibowo E, Pollock PA, Hollis N, Wassersug RJ (2016). Tamoxifen in men: a review of adverse events. Andrology.

[45] Fisher ER, Costantino JP, Leon ME (2007). Pathobiology of small invasive breast cancers without metastases (T1a/b, N0, M0): national surgical adjuvant breast and bowel project (NSABP) protocol B-21. Cancer.

[46] Meattini I, Poortmans PMP, Marrazzo L (2021). Exclusive endocrine therapy or partial breast irradiation for women aged ≥70 years with luminal A-like early stage breast cancer (NCT04134598 - EUROPA): Proof of concept of a randomized controlled trial comparing health related quality of life by patient reported outcome measures. J Geriatr Oncol.

[47] White JR, Anderson SJ, Harris EE (2022). NRG-BR007: a phase III trial evaluating de-escalation of breast radiation (DEBRA) following breast-conserving surgery (BCS) of stage 1, hormone receptor+, HER2-, RS ≤18 breast cancer. J Clin Oncol.

[48] Massarweh SA, Sledge GW, Miller DP, McCullough D, Petkov VI, Shak S (2018). Molecular characterization and mortality from breast cancer in men. J Clin Oncol.

[49] Andre F, Ismaila N, Allison KH (2022). Biomarkers for adjuvant endocrine and chemotherapy in early-stage breast cancer: ASCO guideline update. J Clin Oncol Off J Am Soc Clin Oncol.

