# Supplementary material for: Omission of adjuvant radiotherapy in low-risk elderly males with breast cancer
Source: Breast Cancer. 2024 Mar 20;31(3):485–95. doi: 10.1007/s12282-024-01560-y (PMC11045584; doi:10.1007/s12282-024-01560-y)
Supplement: Supplementary file 1 — Supplementary file1 (DOCX 20 KB) [file 12282_2024_1560_MOESM1_ESM.docx]

**Supplementary Table 1: Descriptive Statistics of Female Cohort**

|  |  | **Adjuvant Therapy** | | | **p** | | |
| --- | --- | --- | --- | --- | --- | --- | --- |
| **Characteristic** | **All patients (N=188683)** | **HT + RT (N=128104)** | **RT (N=25351)** | **HT (N=35228)** | **p-HT + RT vs RT** | **p-HT + RT vs HT** | **p-RT vs HT** |
| Year of Diagnosis (median [range]) | 2012.0 (2004-2019) | 2012.0 (2004-2019) | 2010.0 (2004-2019) | 2013.0 (2004-2019) | <0.001 | <0.001 | <0.001 |
| Age (median [range]) | 73.0 (65-90) | 71.0 (65-90) | 75.0 (65-90) | 78.0 (65-90) | <0.001 | <0.001 | <0.001 |
| Race and Ethnicity |  |  |  |  | <0.001 | <0.001 | <0.001 |
| Asian/Pacific Islander | 3723 (2.0%) | 2711 (2.1%) | 410 (1.6%) | 602 (1.7%) |  |  |  |
| Black | 11331 (6.0%) | 7603 (5.9%) | 1279 (5.0%) | 2449 (7.0%) |  |  |  |
| Hispanic | 5454 (2.9%) | 3781 (3.0%) | 637 (2.5%) | 1036 (2.9%) |  |  |  |
| Native American | 332 (0.2%) | 244 (0.2%) | 30 (0.1%) | 58 (0.2%) |  |  |  |
| Other | 541 (0.3%) | 371 (0.3%) | 70 (0.3%) | 100 (0.3%) |  |  |  |
| Unknown | 1235 (0.7%) | 864 (0.7%) | 172 (0.7%) | 199 (0.6%) |  |  |  |
| White | 166067 (88.0%) | 112530 (87.8%) | 22753 (89.8%) | 30784 (87.4%) |  |  |  |
| Charlson Deyo Score |  |  |  |  | 0.009 | <0.001 | <0.001 |
| 0 | 151212 (80.1%) | 103989 (81.2%) | 20751 (81.9%) | 26472 (75.1%) |  |  |  |
| 1 | 29617 (15.7%) | 19432 (15.2%) | 3655 (14.4%) | 6530 (18.5%) |  |  |  |
| 2+ | 7854 (4.2%) | 4683 (3.7%) | 945 (3.7%) | 2226 (6.3%) |  |  |  |
| Tumor Laterality |  |  |  |  | 0.304 | 0.001 | <0.001 |
| Left | 95074 (50.4%) | 64367 (50.2%) | 12625 (49.8%) | 18082 (51.3%) |  |  |  |
| Right | 93517 (49.6%) | 63678 (49.7%) | 12711 (50.1%) | 17128 (48.6%) |  |  |  |
| Unknown | 92 (0.0%) | 59 (0.0%) | 15 (0.1%) | 18 (0.1%) |  |  |  |
| pT |  |  |  |  | <0.001 | <0.001 | <0.001 |
| T1 | 170773 (90.5%) | 114954 (89.7%) | 23794 (93.9%) | 32025 (90.9%) |  |  |  |
| T2 | 17910 (9.5%) | 13150 (10.3%) | 1557 (6.1%) | 3203 (9.1%) |  |  |  |
| Tumor Size (median [range]) | 11.0 (0-30) | 11.0 (0-30) | 9.0 (1-30) | 11.0 (0-30) | <0.001 | <0.001 | <0.001 |
| Histology |  |  |  |  | <0.001 | <0.001 | <0.001 |
| IDC | 137252 (72.7%) | 93482 (73.0%) | 18540 (73.1%) | 25230 (71.6%) |  |  |  |
| ILC | 18818 (10.0%) | 13478 (10.5%) | 1912 (7.5%) | 3428 (9.7%) |  |  |  |
| Other | 32613 (17.3%) | 21144 (16.5%) | 4899 (19.3%) | 6570 (18.6%) |  |  |  |
| ER |  |  |  |  | <0.001 | 0.004 | <0.001 |
| Negative | 646 (0.3%) | 327 (0.3%) | 263 (1.0%) | 56 (0.2%) |  |  |  |
| Positive | 188001 (99.6%) | 127754 (99.7%) | 25080 (98.9%) | 35167 (99.8%) |  |  |  |
| Unknown | 36 (0.0%) | 23 (0.0%) | 8 (0.0%) | 5 (0.0%) |  |  |  |
| PR |  |  |  |  | <0.001 | 0.817 | <0.001 |
| Negative | 20526 (10.9%) | 13487 (10.5%) | 3325 (13.1%) | 3714 (10.5%) |  |  |  |
| Positive | 167327 (88.7%) | 114084 (89.1%) | 21867 (86.3%) | 31376 (89.1%) |  |  |  |
| Unknown | 830 (0.4%) | 533 (0.4%) | 159 (0.6%) | 138 (0.4%) |  |  |  |
| HER2 |  |  |  |  | <0.001 | <0.001 | <0.001 |
| Negative | 124335 (65.9%) | 84852 (66.2%) | 12995 (51.3%) | 26488 (75.2%) |  |  |  |
| Positive | 3563 (1.9%) | 2307 (1.8%) | 460 (1.8%) | 796 (2.3%) |  |  |  |
| Unknown | 60785 (32.2%) | 40945 (32.0%) | 11896 (46.9%) | 7944 (22.6%) |  |  |  |
| Grade |  |  |  |  | <0.001 | <0.001 | <0.001 |
| Grade 1 | 70916 (38.8%) | 46326 (36.2%) | 11046 (43.6%) | 13544 (38.4%) |  |  |  |
| Grade 2 | 86825 (47.5%) | 60779 (47.4%) | 10482 (41.3%) | 15564 (44.2%) |  |  |  |
| Grade 3 | 16558 (9.1%) | 11667 (9.1%) | 2203 (8.7%) | 2688 (7.6%) |  |  |  |
| Unknown | 8585 (4.7%) | 5721 (4.5%) | 1277 (5.0%) | 1587 (4.5%) |  |  |  |
| LVSI |  |  |  |  | <0.001 | <0.001 | <0.001 |
| LVSI+ | 6515 (3.5%) | 4670 (3.6%) | 528 (2.1%) | 1317 (3.7%) |  |  |  |
| LVSI- | 110093 (58.3%) | 74514 (58.2%) | 11778 (46.5%) | 23801 (67.6%) |  |  |  |
| Unknown | 72075 (38.2%) | 48920 (38.2%) | 13045 (51.5%) | 10110 (28.7%) |  |  |  |

*HT* hormone therapy, *RT* radiation therapy, *IDC* intraductal carcinoma, *ILC* intralobular carcinoma, *ER* estrogen receptor, *PR* progesterone receptor, *HER2* human epidermal growth receptor 2, *LVSI* lymphovascular space invasion

**Supplemental Table 2: Multivariable Cox Proportional-Hazards Regression of OS in Female Cohort**

|  | **Unadjusted** | | **IPTW** | |
| --- | --- | --- | --- | --- |
| **Category** | **HR** | **p** | **HR** | **p** |
| Age | 1.092 (1.069-1.116) | <0.001 | 1.108 (1.079-1.137) | <0.001 |
| **Charlson Deyo Score** |  |  |  |  |
| 0 | Ref |  | Ref |  |
| 1 | 1.215 (0.818-1.804) | 0.336 | 1.795 (1.074-2.999) | 0.026 |
| 2+ | 1.933 (0.996-3.752) | 0.051 | 2.821 (0.945-8.42) | 0.063 |
| **pT** |  |  |  |  |
| T1 | Ref |  | Ref |  |
| T2 | 1.204 (0.794-1.824) | 0.382 | 0.827 (0.438-1.562) | 0.559 |
| **Grade** |  |  |  |  |
| Grade 1 | Ref |  | Ref |  |
| Grade 2 | 0.984 (0.721-1.343) | 0.921 | 1.052 (0.692-1.6) | 0.812 |
| Grade 3 | 1.146 (0.671-1.957) | 0.617 | 1.376 (0.676-2.797) | 0.379 |
| Unknown | 0.639 (0.258-1.585) | 0.334 | 0.608 (0.283-1.306) | 0.202 |
| **Adjuvant Therapy** |  |  |  |  |
| HT | Ref |  | Ref |  |
| HT + RT | 0.59 (0.408-0.853) | 0.005 | 0.808 (0.533-1.225) | 0.315 |
| RT | 0.474 (0.285-0.789) | 0.004 | 0.786 (0.448-1.38) | 0.402 |
